# Regional differences in the temporal evolution of stroke: a population-based study of Brazil according to sex in individuals aged 15–49 years between 1997 and 2012

**DOI:** 10.1186/s13104-018-3439-x

**Published:** 2018-05-21

**Authors:** Laércio da Silva Paiva, Jean Henri Maselli Schoueri, Luiz Vinicius de Alcantara Sousa, Rodrigo Daminello Raimundo, Erika da Silva Maciel, João Antonio Correa, Fernando Adami

**Affiliations:** 1 Departamento de Saúde da Coletividade, Laboratório de Epidemiologia e Análise de dados, Faculdade de Medicina do ABC, Av. Lauro Gomes, 2000, Vila Sacadura Cabral, Santo André, SP CEP: 09060-870 Brazil; 2 Departamento de Saúde da Coletividade, Laboratório de Delineamento de Estudos e Escrita Científica, Faculdade de Medicina do ABC, Av. Lauro Gomes, 2000, Vila Sacadura Cabral, Santo André, SP CEP: 09060-870 Brazil; 3 Universidade Federal do Tocantis, Avenida Lourdes Solino s/n°-Setor Universitário, Miracema, TO Brazil; 4 Disciplina de Angiologia e Cirurgia Vascular, Faculdade de Medicina do ABC, Av. Lauro Gomes, 2000, Vila Sacadura Cabral, Santo André, SP CEP: 09060-870 Brazil

**Keywords:** Stroke, Brazil, Epidemiology, Mortality, Temporal trend

## Abstract

**Objective:**

The present study analyzed the temporal trend of stroke mortality according to sex in individuals aged 15**–**49 years in the different regions of Brazil between 1997 and 2012.

**Results:**

There was progressive reduction in mortality rate due to stroke in Brazil. The reduction trend was the same for both sexes, although mortality remained slightly higher among men. There was a difference in mortality rates according to the administrative region of the country.

**Electronic supplementary material:**

The online version of this article (10.1186/s13104-018-3439-x) contains supplementary material, which is available to authorized users.

## Introduction

Stroke is among the second and third leading causes of death worldwide [1]. It is estimated that across a year, 15 million people experience stroke and about one-third of remains impaired [2]. In Brazil, the proportion is the same: death following stroke only follows deaths due to acute myocardial infarction (AMI) and pneumonia [3].

Among young people, stroke is less common [4], however, in recent years the incidence and risk factors have increased in this population [5]. It has been noted that the increase in the incidence of stroke is higher in women under 30 years of age than in men under 30 years of age [4]. There is a divergence in the literature regarding the magnitude of stroke mortality between the sexes [6–9], although data from the Institute for Health Metrics and Evaluation (2016) show that the overall mortality rate from stroke is higher in females [10].

In addition, studies have related the risk of stroke with other factors, for example socioeconomic conditions, and have demonstrated the frequency of occurrence and consequences of stroke in different administrative regions of Brazil [11, 12]. It should be emphasized that the consequences of stroke in young adults are catastrophic due to the limitations it causes in the period of high productivity [1].

The question arises as to the applicability of the international findings to the Brazilian context. Thus, the present study aims to describe the temporal trend of mortality due to stroke in individuals aged 15–49 years, stratified by sex in the different regions of Brazil between 1997 and 2012.

## Main text

### Methods

This was an ecological study in Brazil, which evaluated the temporal trend of stroke mortality in young Brazilian adults aged 15–49 years, from 1997 to 2012, taking into account the sex of individuals and the region of the country, by means of a secondary analysis of data. Data were collected from the Department of Informatics of the Unified Health System (DATASUS), an agency of the Ministry of Health that includes a national database and is considered an important management tool in Brazilian healthcare [13].

The study considered all deaths recorded by the Mortality Information System (SIM) for stroke in men and women aged 15–49 years and took regions of Brazil into account. The period studied was January 1, 1997 to December 31, 2012. Data from the SIM were used and was publicly available and unrestricted by the DATASUS website (http://www.datasus.gov.br). According to the most recent report, data coverage has been improving across the last decade, reaching 96.1% in Brazil in 2011 [14].

Stroke was defined according to the tenth revision of the International Classification of Diseases (ICD10) codes: I60 (subarachnoid hemorrhage), I61 (intracranial hemorrhage), I63 (cerebral infarction) and I64 (stroke not specified between ischemic or hemorrhagic) [15].

All DATASUS data selection was performed by two independent researchers using extraction sheets designed by the authors; a third researcher was responsible for correcting any discrepancies found. The mortality calculation was performed by dividing the number of deaths by stroke in young adults, stratified by sex and administrative region of Brazil, by the total population at risk and then multiplied by 100,000.

Stroke mortality was standardized by age using the direct method, with the World Health Organization (WHO) standard population as the reference population [16].

The percentage change (PC) and the annual percentage change (APC) are the two trend measures in this analysis. For the calculation of the PC, the initial amount of the same rate was subtracted from the final value of the adjusted rate for stroke mortality, dividing the result by the initial value of the rate and multiplied by 100. The calculation was repeated for each region and according to sex. The APC, on the other hand, was calculated using the angular coefficient (β), derived from the linear regression as shown by Fay et al. [17].

The data used in this project come from a public database of national scope, which is unrestricted and allows public access. This means that there is no need for evaluation by the Research Ethics Committee according to the Resolution of the National Health Council (CNS) of no 466 of December 12, 2012.

#### Statistical analysis

To describe the mortality rate of stroke, the relative and absolute frequencies were used. Linear regression was applied to compare temporal trends, with calendar years as the independent variable, and mortality as the dependent variable. We estimated the slope (β), its respective probability (p), and predictive capacity of the model (r^2^). The level of significance was set at 5%. The statistical program used was *Stata*, version 11.0.

### Results

In Brazil, between 1997 and 2012, there were 62,751 deaths in men and 62,115 deaths in women between the ages of 15 and 49 (Table 1).

**Table 1:** Deaths, PC and APC of stroke in men and women aged 15–49 between 1997 and 2012

Characteristics	Men			Women		
Regions	Deaths	PC	APC (95% CI)	Deaths	PC	APC (95% CI)
North	3663	− 19.73	− 0.019 (− 0.029; − 0.008)*	3520	− 27.59	− 0.025 (− 0.034; − 0.016)*
Northeast	14,025	− 20.00	− 0.022 (− 0.029; − 0.015)*	14,763	− 35.16	− 0.027 (− 0.035; − 0.020)*
Southeast	32,128	− 58.54	− 0.061 (− 0.066; − 0.055)*	30,931	− 54.74	− 0.054 (− 0.059; − 0.050)*
South	8624	− 58.33	− 0.056 (− 0.064; − 0.049)*	8609	− 57.14	− 0.059 (− 0.066; − 0.052)*
Midwest	4311	− 57.77	− 0.053 (− 0.060; − 0.045)*	4292	− 53.78	− 0.057 (− 0.064; − 0.049)*
Brazil	62,751	− 50.38	− 0.049 (− 0.054; − 0.044)*	62,115	− 49.57	− 0.048 ( − 0.513; − 0.446)*

*PC* percent change, *APC* annual percent change, *95% CI* 95% Confidence Interval

* Statistically significant difference

In Table 1 it can be observed that the Annual Percentage Change (APC) of the age-standardized mortality rate is negative throughout the country, with the predominance of reductions in the South, APC = − 0.056 (CI 95%  − 0.064; 0.049) for men and  − 0.059 (CI 95%  − 0.066;  − 0.052) for women; and in the Southeast region, APC = − 0.061 (CI 95%  − 0.066;  − 0.055) for the male population and  − 0.054 (CI 95%  − 0.059;0.050) for the female population.

**Fig. 1:**
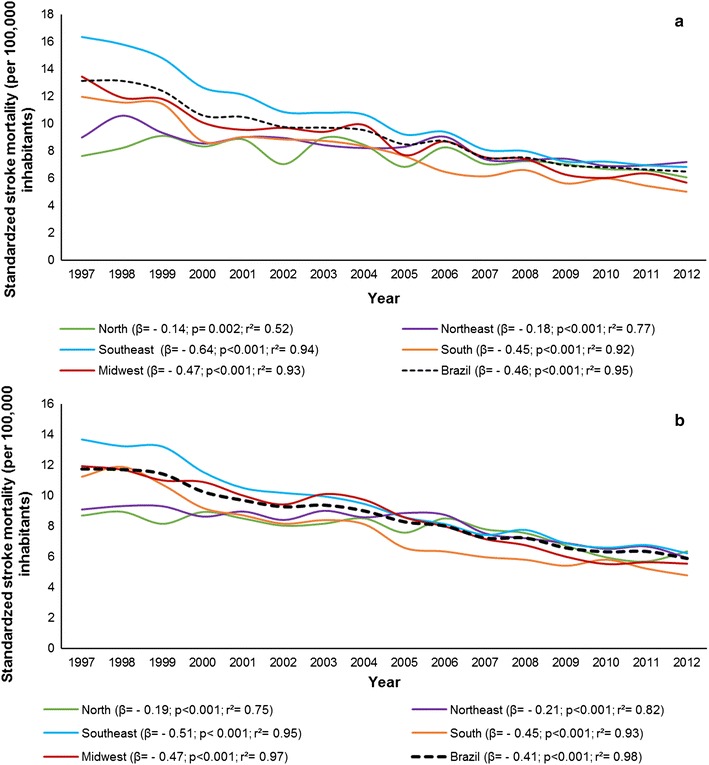
Age-standardized mortality rate for stroke (100,000 inhabitants) among men (**a**) and women (**b**), resident in Brazil aged 15–49 years, in the period from 1997 to 2012 and estimates obtained from linear regression, according to year and regions

In addition, there was a progressive decrease in the mortality rate due to age-standardized stroke in Brazil. There is similarity for both sexes, but mortality is slightly higher among men (Fig. 1).

There was a difference in mortality rates according to the country’s administrative region. The Southeast region presented the largest reduction in deaths due to stroke in men (β =  − 0.64; p < 0.001; r^2^ = 0.94) and for women (β =  − 0.51, p < 0. 001; r^2^ = 0.95); and the North region presented the lowest risk reduction for stroke in men (β =  − 0.14; p = 0.002; r^2^ = 0.52) and women (β =  − 0.19; p < 0.001; r^2^ = 0.75) (Fig. 1 and Additional file 1).

When stratifying by regions of Brazil and age groups, between 1997 and 2012, there is a significant reduction in the mortality rate due to stroke in the Northern region in the age range 30–39 and 40–49 years in men and in the age groups of 20–29 and 40–49 years in women. In the Northeastern and Southern regions, the reduction in mortality due to stroke occurs in the age range between 20 and 49 years for both sexes. In the Southeast and Central-West regions, this decrease occurs in all age groups, both for men and women, except for the men of 15–19 years of the Midwest (Table 2).

**Table 2:** Linear regression of mortality†* by stroke (per 100,000 inhabitants) of Brazilians aged 15–49 years, between 1997 and 2012, according to the regions of Brazil stratified by sex Source: Mortality Information System (SIM) obtained by the Information Department of the Brazilian National Health System (DATASUS)

Country regions	Linear regression mortality			
	Men		Women	
	β (95% CI)	p	β (95% CI)	p
Age group (years)				
North				
15–19	− 0.004 (− 0.020; 0.042)	0.841	− 0.013 (− 0.045; 0.019)	0.402
20–29	− 0.002 (− 0.068; 0.063)	0.945	− 0.034 (− 0.063; − 0.005)	0.024
30–39	− 0.110 (− 0.167; − 0.052)	0.001	− 0.072 (− 0.157; 0.012)	0.090
40–49	− 0.399 (− 0.685; − 0.113)	0.010	− 0.635 (− 0.843; − 0.427)	< 0.001
Northeast				
15–19	0.009 (− 0.020; 0.039)	0.500	− 0.010 (− 0.025; 0.003)	0.139
20–29	− 0.047 (− 0.093; − 0.001)	0.045	− 0.054 (− 0.084; − 0.024)	0.002
30–39	− 0.149 (− 0.207; − 0.092)	< 0.001	− 0.228 (− 0.269; − 0.188)	< 0.001
40–49	− 0.497 (− 0.685; − 0.309)	< 0.001	− 0.522 (− 0.755; − 0.289)	< 0.001
Southeast				
15–19	− 0.040 (− 0.068; − 0.012)	0.008	− 0.045 (− 0.069; −0.021)	0.001
20–29	− 0.058 (− 0.094; − 0.022)	0.004	− 0.093 (− 0.131; − 0.056)	< 0.001
30–39	− 0.478 (− 0.574; − 0.383)	< 0.001	− 0.475 (− 0.556; − 0.394)	< 0.001
40–49	− 1.889 (− 2.138; − 1.640)	< 0.001	− 1.360 (− 1.501; − 1.218)	< 0.001
South				
15–19	− 0.010 (− 0.044; 0.024)	0.528	0.005 (− 0.019; 0.029)	0.670
20–29	− 0.034 (− 0.069; − 0.001)	0.047	− 0.080 (− 0.121; − 0.040)	0.001
30–39	− 0.310 (− 0.387; − 0.233)	< 0.001	− 0.339 (− 0.422; − 0.256)	< 0.001
40–49	− 1.365 (− 1.571; − 1.159)	< 0.001	− 1.333 (− 1.549; − 1.116)	< 0.001
Central-West				
15–19	− 0.008 (− 0.047; 0.031)	0.666	− 0.047 (− 0.082; − 0.012)	0.012
20–29	− 0.062 (− 0.099; − 0.025)	0.003	− 0.098 (− 0.144; − 0.052	< 0.001
30–39	− 0.377 (− 0.508; − 0.247)	< 0.001	− 0.378 (− 0.456; − 0.301)	< 0.001
40–49	− 1.302 (− 1.539; − 1.065)	< 0.001	− 1.280 (− 1.504; − 1.055)	< 0.001

*β* regression slope, *95% CI* 95% confidence interval

†Crude rate. * International Statistical Classification of Diseases and Related Health Problems, 10th revision: I60, I61, I63 to I64 [15]

### Discussion

Cerebrovascular diseases are the leading cause of mortality in women and the second in men in industrialized countries [18]. In this context, stroke should be highlighted as, besides being an important cause of functional impairment, it is expected that associated mortality rates will increase, reaching 7.8 million deaths by 2030 [19–21].

However, the situation is different for young adults, in an American study based on secondary data, a decrease in mortality due to stroke was observed in young adults suggesting that there was improvement in the recognition and treatment of the disease in the last two decades [22].

Global data [10] indicate a reduction in the mortality rate due to stroke in young adults, in men 14.01 (95% CI 13.17; 15.30) in 1990 to 12.57 (95% CI 12.07; 13.06) in 2016 and in women 10.83 (95% CI 10.14; 11.67) in 1990 to 7.44 (95% CI 7.13; 7.75) in 2016.

Similarly, in a national study, a reduction in global stroke mortality in Brazil became evident [12, 23] among young adults, which is in agreement with our results. Both results indicate that this reduction has been occurring for years in both sexes.

The results from this study regarding may be justified, in part, by greater care in modifying and treating vascular risk factors, especially regarding modifiable risk factors such as hypertension, diabetes, dyslipidemia, a sedentary lifestyle, and smoking [24]. In addition, one cannot forget that men and women have different physiologies. In this context, the guidelines already show the importance of risk factors for stroke that are more commonly—or exclusively—found in the female population, such as migraine with aura, depression, diabetes mellitus, and a history of complications in pregnancy [25].

Additionally, it is necessary to consider a possible association between socioeconomic status and the risk for stroke. In a national study, it was concluded that income inequity should be considered as a determinant health factor in less well-off patients [26]. The same study also indicated that the participation of each state in the financing of the health system has a negative impact on stroke mortality in the poorest regions of the North and Northeast of Brazil over time; at the same time, the highest expenditure of states regarding health was related to increased stroke mortality [26].

Brazil is divided into five regions, North, Northeast, Southeast, South and Midwest. The Southeast region has the largest number of inhabitants and the North region the smallest of the country [27].

It should be taken into account that the populations of the North and Northeast of Brazil present a lower rate of urbanization concomitant with higher rates of child dependence and mortality, among other socioeconomic indicators that can be interpreted as markers of development of a region. Comparatively, the Southeast region, as well as the South region, have the highest monthly family incomes per capita in Brazil, which may explain why the least developed regions (North and Northeast) present lower reductions in the mortality rate due to stroke in young adults [28].

After all, given that higher incidence and mortality rates can be seen in low-and middle-income countries when compared to high-income countries, we could extrapolate to regions of the same country that present different levels of development in order to understand the results found [29].

Thus, along with the above mentioned reasons and, similar to what occurs with several other diseases with serious consequences for the population, the prevention of stroke is paramount. After all, if prevention is done correctly, it avoids both the event and its direct or indirect complications that may be related to slower recovery and worse functional results, along with affecting mortality rates [30, 31].

In this context, the control of risk factors is essential for the prevention of stroke [32], especially as some modifiable risk factors account for about 90% of the risks attributed to the stroke population [33]. Moreover, there are high hospital costs related to stroke, especially in the first year after stroke [34].

Thus, it is necessary to carry out new studies to understand the temporal trends of the risk factors for stroke in these regions of Brazil that over time may change the scenario of this young adult population.

### Conclusion

Stroke mortality has declined in Brazil in all regions of the country for both young men and women; the reduction is slightly higher among men. The Southeast region showed the highest declines and the North region showed the lowest, in both sexes.

### Limitations

The present study is a secondary data analysis and therefore there is a difference between the amount of cases and their respective registration, apart from an inequality between the number of cases registered by region. More than conjecture, this is supported by the uncertainty of the data presented in some states of the North and Northeast regions. This would restrict the usefulness of their data, especially as a result of the inconsistencies in comparison with data from states and capital municipalities [35] or in comparison with the data coverage of the other Brazilian regions [14].

In addition, stratification by Stroke subtype was not possible, as approximately one-third of the deaths recorded in DATASUS refer to the “not specified” (NE) category, in other words indicating either ischemic or hemorrhagic stroke.

## Additional file

**Additional file 1.** Standardized mortality estimated for standardized stroke (per 100,000 population) between men and women, residing in Brazil aged 15 to 49 years, from 1997 to 2012 and estimates obtained from linear regression, second year and regions.

## Abbreviations

AMI: acute myocardial infarction
DATASUS: Department of Informatics of the Unified Health System
SIM: Mortality Information System
ICD10: International Classification of Diseases
WHO: World Health Organization
PC: percentage change
APC: annual percentage change
CI 95%: 95% confidence interval
