# Supplementary material for: Regional differences in the temporal evolution of stroke: a population-based study of Brazil according to sex in individuals aged 15–49 years between 1997 and 2012
Source: BMC Res Notes. 2018 May 21;11:326. doi: 10.1186/s13104-018-3439-x (PMC5963170; doi:10.1186/s13104-018-3439-x)
Supplement: Supplementary file 1 — Additional file 1. Standardized mortality estimated for standardized stroke (per 100,000 population) between men and women, residing in Brazil aged 15 to 49 years, from 1997 to 2012 and estimates obtained from linear regression, second year and regions. [file 13104_2018_3439_MOESM1_ESM.docx]

**Additional file 1.**

| **Brazil/Regions** | **Stroke Mortality* (x100,000 inhabitants)** | | | | | | | | | | | | | | | | **Linear Regression** | | |
| --- | --- | --- | --- | --- | --- | --- | --- | --- | --- | --- | --- | --- | --- | --- | --- | --- | --- | --- | --- |
|  | **Men** | | | | | | | | | | | | | | | |  |  |  |
|  | **1997** | **1998** | **1999** | **2000** | **2001** | **2002** | **2003** | **2004** | **2005** | **2006** | **2007** | **2008** | **2009** | **2010** | **2011** | **2012** | β | p - value | r² |
| **Brazil** | 13.1 (13.1 - 13.2) | 13.1 (13.1 - 13.2) | 12.4 (12.4 - 12.4) | 10.6 (10.6 - 10.6) | 10.5 (10.5 - 10.5) | 9.8 (9.8 - 9.8) | 9.7 (9.7 - 9.7) | 9.5 (9.5 - 9.6) | 8.5 (8.5 - 8.5) | 8.8 (8.8 - 8.8) | 7.5 (7.5 - 7.5) | 7.5 (7.5 - 7.6) | 7.0 (7.0 - 7.0) | 6.8 (6.8 - 6.8) | 6.7 (6.7 - 6.7) | 6.5 (6.5 - 6.5) | -0.46 | <0.001 | 0.95 |
| **North** | 7.6 (7.6 - 7.7) | 8.2 (8.2 - 8.2) | 9.1 (9.1 - 9.1) | 8.3 (8.3 - 8.4) | 8.9 (8.8 - 8.9) | 7.0 (7.0 - 7.1) | 9.0 (8.9 - 9.0) | 8.5 (8.4 - 8.5) | 6.8 (6.8 - 6.9) | 8.3 (8.2 - 8.3) | 7.0 (7.0 - 7.1) | 7.3 (7.2 - 7.3) | 7.0 (7.0 - 7.1) | 6.7 (6.6 - 6.7) | 6.6 (6.6 - 6.6) | 6.1 (6.0 - 6.1) | -0.14 | 0.002 | 0.52 |
| **Northeast** | 9.0 (9.0 - 9.0) | 10.6 (10.6 - 10.6) | 9.3 (9.3 - 9.4) | 8.6 (8.6 - 8.6) | 9.0 (9.0 - 9.0) | 9.0 (9.0 - 9.0) | 8.5 (8.4 - 8.5) | 8.2 (8.2 - 8.3) | 8.3 (8.3 - 8.3) | 9.1 (9.1 - 9.1) | 7.4 (7.4 - 7.4) | 7.4 (7.4 - 7.4) | 7.5 (7.5 - 7.5) | 6.9 (6.9 - 7.0) | 7.0 (7.0 - 7.0) | 7.2 (7.2 - 7.2) | -0.18 | <0.001 | 0.77 |
| **Southeast** | 16.4 (16.4 - 16.4) | 15.8 (15.8 - 15.9) | 14.8 (14.8 - 14.8) | 12.7 (12.7 - 12.7) | 12.1 (12.1 - 12.1) | 10.9 (10.9 - 10.9) | 10.8 (10.8 - 10.8) | 10.7 (10.7 - 10.7) | 9.2 (9.2 - 9.2) | 9.4 (9.4 - 9.4) | 8.1 (8.1 - 8.1) | 8.0 (8.0 - 8.0) | 7.2 (7.2 - 7.3) | 7.2 (7.2 - 7.2) | 7.0 (7.0 - 7.0) | 6.8 (6.8 - 6.9) | -0.64 | <0.001 | 0.94 |
| **South** | 12.0 (12.0 - 12.0) | 11.6 (11.6 - 11.6) | 11.5 (11.5 - 11.5) | 8.7 (8.7 - 8.8) | 9.1 (9.0 - 9.1) | 8.9 (8.9 - 8.9) | 8.8 (8.8 - 8.8) | 8.4 (8.4 - 8.4) | 7.7 (7.6 - 7.7) | 6.5 (6.5 - 6.5) | 6.2 (6.2 - 6.2) | 6.6 (6.6 - 6.6) | 5.6 (5.6 - 5.7) | 6.0 (6.0 - 6.0) | 5.5 (5.5 - 5.5) | 5.0 (5.0 - 5.1) | -0.45 | <0.001 | 0.92 |
| **Midwest** | 13.5 (13.4 - 13.5) | 11.9 (11.9 - 12.0) | 11.8 (11.8 - 11.9) | 10.1 (10.1 - 10.1) | 9.6 (9.5 - 9.6) | 9.7 (9.7 - 9.7) | 9.4 (9.4 - 9.4) | 9.9 (9.9 - 10.0) | 7.7 (7.7 - 7.7) | 8.7 (8.7 - 8.7) | 7.5 (7.5 - 7.6) | 7.4 (7.4 - 7.5) | 6.3 (6.3 - 6.3) | 6.0 (6.0 - 6.1 ) | 6.4 (6.3 - 6.4) | 5.7 (5.7 - 5.7) | -0.47 | <0.001 | 0.93 |
| **Brazil/Regions** | **Women** | | | | | | | | | | | | | | | | **Linear Regression** | | |
|  | **1997** | **1998** | **1999** | **2000** | **2001** | **2002** | **2003** | **2004** | **2005** | **2006** | **2007** | **2008** | **2009** | **2010** | **2011** | **2012** | β | p - value | r² |
| **Brazil** | 11.7 (11.7 - 11.7) | 11.7 (11.7 - 11.7) | 11.4 (11.4 - 11.4) | 10.2 (10.2 - 10.3) | 9.7 (9.7 - 9.7) | 9.3 (9.2 - 9.3) | 9.4 (9.4 - 9.4) | 9.0 (9.0 - 9.0) | 8.3 (8.3 - 8.3) | 8.0 (8.0 - 8.0) | 7.2 (7.2 - 7.2) | 7.2 (7.2 - 7.2) | 6.6 (6.6 - 6.6) | 6.3 (6.3 - 6.3) | 6.3 (6.3 - 6.3) | 5.9 (5.9 - 5.9) | -0.41 | <0.001 | 0.98 |
| **North** | 8.7 (8.7 - 8.7) | 8.9 (8.9 - 9.0) | 8.1 (8.1 - 8.2) | 8.9 (8.9 - 9.0) | 8.5 (8.5 - 8.5) | 8.0 (8.0 - 8.0) | 8.2 (8.1 - 8.2) | 8.5 (8.5 - 8.5) | 7.6 (7.5 - 7.6) | 8.5 (8.5 - 8.5) | 7.8 (7.8 - 7.8) | 7.5 (7.5 - 7.6) | 6.7 (6.7 - 6.7) | 5.9 (5.9 - 6.0) | 5.7 (5.7 - 5.7) | 6.3 (6.3 - 6.4) | -0.19 | <0.001 | 0.75 |
| **Northeast** | 9.1 (9.1 - 9.1) | 9.3 (9.3 - 9.3) | 9.3 (9.3 - 9.3) | 8.6 (8.6 - 8.6) | 9.0 (8.9 - 9.0) | 8.4 (8.4 - 8.4) | 9.0 (9.0 - 9.0) | 8.6 (8.6 - 8.6) | 8.9 (8.9 - 8.9) | 8.7 (8.7 - 8.8) | 7.5 (7.5 - 7.5) | 7.2 (7.2 - 7.2) | 6.9 (6.9 - 6.9) | 6.5 (6.5 - 6.5) | 6.7 (6.6 - 6.7) | 5.9 (5.9 - 5.9) | -0.21 | <0.001 | 0.82 |
| **Southeast** | 13.7 (13.6 - 13.7) | 13.2 (13.2 - 13.2) | 13.2 (13.2 - 13.2) | 11.5 (11.5 - 11.6) | 10.5 (10.5 - 10.5) | 10.2 (10.2 - 10.2) | 9.9 (9.9 - 10.0) | 9.4 (9.4 - 9.5) | 8.6 (8.6 - 8.6) | 8.1 (8.1 - 8.1) | 7.4 (7.4 - 7.4) | 7.7 (7.7 - 7.8) | 6.9 (6.9 - 6.9) | 6.6 (6.6 - 6.6) | 6.8 (6.7 - 6.8) | 6.2 (6.2 - 6.2) | -0.51 | <0.001 | 0.95 |
| **South** | 11.2 (11.2 - 11.3) | 11.9 (11.9 - 11.9) | 10.7 (10.7 - 10.7) | 9.2 (9.2 - 9.2) | 8.7 (8.7 - 8.7) | 8.2 (8.1 - 8.2) | 8.4 (8.4 - 8.4) | 8.1 (8.1 - 8.2) | 6.6 (6.6 - 6.6) | 6.4 (6.3 - 6.4) | 6.0 (6.0 - 6.0) | 5.8 (5.8 - 5.8) | 5.4 (5.4 - 5.4) | 5.8 (5.8 - 5.8) | 5.2 (5.2 - 5.2) | 4.8 (4.8 - 4.8) | -0.45 | <0.001 | 0.93 |
| **Midwest** | 11.9 (11.9 -12.0) | 11.7 (11.7 - 11.7) | 11.0 (11.0 - 11.0) | 10.9 (10.9 - 10.9) | 10.0 (10.0 - 10.0) | 9.4 (9.4 - 9.4) | 10.1 (10.1 - 10.1) | 9.7 (9.7 - 9.8) | 8.6 (8.5 - 8.6) | 8.0 (7.9 - 8.0) | 7.1 (7.1 - 7.2) | 6.7 (6.7 - 6.8) | 6.0 (6.0 - 6.0) | 5.5 (5.5 - 5.5) | 5.6 (5.6 - 5.7) | 5.5 (5.5 - 5.6) | -0.47 | <0.001 | 0.98 |

**Additional file 1.** Standardized mortality estimated for standardized stroke^†^ (per 100,000 population) between men and women, residing in Brazil aged 15 to 49 years, from 1997 to 2012 and estimates obtained from linear regression, second year and regions.

^†^ Standardized by the World Health Organization standard population. [16] * International Statistical Classification of Diseases and Related Health Problems, 10th revision: I60, I61, I63 to I64. [15]

Source: Mortality Information System (SIM) obtained by the Information Department of the Brazilian National Health System (DATASUS).
